# Effects of curcumin on depression, anxiety, and stress symptoms in adults: a GRADE-assessed systematic review and dose–response meta-analysis

**DOI:** 10.29219/fnr.v70.14735

**Published:** 2026-09-24

**Authors:** Saleh A. Alsanie, Mansour Alblaji, Nouf Abdullah Alharbi, Fahad Saad Alhodieb, Moein Askarpour

**Affiliations:** 1 Department of Basic Health Sciences, College of Applied Medical Sciences, Qassim University, Buraydah, Saudi Arabia; 2 Department of Clinical Nutrition, Medical City, Qassim University, Buraydah, Saudi Arabia; 3 Social Determinants of Health Research Center, Institute for Futures Studies in Health, Kerman University of Medical Sciences, Kerman, Iran

**Keywords:** curcumin, depressive disorder, anxiety disorders, stress, meta-analysis as topic

## Abstract

**Background:**

Curcumin, a bioactive polyphenol extracted from *Curcuma longa*, has demonstrated antioxidant, anti-inflammatory, and neuroprotective properties that may benefit mental health. However, evidence from randomized controlled trials (RCTs) remains inconsistent.

**Objective:**

This systematic review and dose–response meta-analysis aimed to quantitatively assess the effects of curcumin supplementation on depression, anxiety, and stress in adults.

**Methods:**

Electronic databases including PubMed, Scopus, Web of Science, and Google Scholar were searched up to December 2025 for RCTs. Two reviewers independently conducted study selection, data extraction, and risk-of-bias assessment. Pooled standardized mean differences (SMDs) with 95% confidence intervals (CIs) were calculated. Subgroup, meta-regression, sensitivity, dose–response, and time–response analyses were conducted. Moreover, publication bias and certainty of evidence were evaluated. The study protocol was registered in the PROSPERO database (CRD420251208049).

**Results:**

Twenty-six RCTs encompassing 1,491 participants were included. Curcumin supplementation significantly reduced symptoms of depression (SMD = –1.53; 95% CI: –2.05 to –1.02), anxiety (SMD = –1.67; 95% CI: –2.42 to –0.93), and stress (SMD = –1.71; 95% CI: –3.37 to –0.06) compared with placebo. Dose–response analysis indicated a significant nonlinear association, suggesting that higher curcumin doses (>1.5 g/day) were associated with greater reductions in depressive symptoms. Subgroup analyses showed slightly greater improvements among female participants and those experiencing psychological distress.

**Conclusions:**

Curcumin supplementation may provide a potential benefit for depression, anxiety, and stress; however, substantial between-study heterogeneity and moderate-to-low certainty of evidence limit confidence in the generalizability of these findings. Further well-designed RCTs are warranted to confirm these findings.

## Popular scientific summary

- Curcumin supplementation significantly reduced symptoms of depression, anxiety, and stress.
- Dose–response analysis revealed greater improvements with higher curcumin doses.
- Evidence was synthesized from 26 RCTs with 1,491 adults across varied populations and formulations.
- Curcumin may have potential benefits for mental well-being; however, the available evidence is limited by substantial heterogeneity and moderate-to-low certainty.

Psychological disorders, particularly depression, anxiety, and stress, are among the most prevalent and disabling mental health conditions worldwide. According to the Global Burden of Disease 2025 report, in 2021, there were an estimated 359 million anxiety and 332 million depression cases globally, with the greatest burden observed in younger individuals and populations from low- and middle-income regions. By 2040, the global prevalence of anxiety and depressive disorders is expected to exceed 515 million and 466 million, respectively, emphasizing their escalating public health burden (1).

Despite the central role of pharmacological interventions – such as selective serotonin and serotonin–norepinephrine reuptake inhibitors – and cognitive-behavioral therapy in managing these disorders, their effectiveness is often limited, and issues like side effects and poor adherence are common. As a result, increasing interest has emerged in alternative and adjunctive therapies that offer better tolerability and potential clinical benefits (2, 3).

Curcumin is a bioactive polyphenol extracted from the root of *Curcuma longa* (turmeric) that has been widely investigated for its pleiotropic biological activities. Due to its potent anti-inflammatory, antioxidant, and neuroprotective properties, curcumin has long held an important place in traditional Ayurvedic and Chinese medicine (4). In recent years, it has gained substantial scientific attention as a promising bioactive compound with potential to improve both physical and mental health. Its broad spectrum of biological actions suggests that curcumin may play a meaningful role in preventing and managing chronic disorders, particularly those involving metabolic and cardiovascular dysfunction (5–9). Moreover, its potential effects on mental health are thought to occur through mechanisms such as reducing inflammation, modulating neurotransmitter levels, and decreasing oxidative stress (10).

Several randomized controlled trials (RCTs) have examined the psychotropic effects of curcumin, but findings have been inconsistent. Moreover, previous meta-analyses (11–14), which varied in design and focus, suggested benefits for depression and anxiety; however, due to the lack of dose– and time–response analyses, limited study numbers, and the absence of simultaneous evaluation of depression, anxiety, and stress, their conclusions remain limited.

Given the growing number of RCTs on curcumin’s psychotropic effects and the limitations of previous reviews, an updated and comprehensive meta-analysis covering key mental health outcomes, including depression, anxiety, and stress, is warranted. This study therefore aimed to systematically review and quantitatively synthesize the current evidence on the effects of curcumin supplementation on indices of psychological well-being in adults, providing a clear and integrated overview of its potential benefits.

## Methods

This systematic review and meta-analysis followed the Preferred Reporting Items for Systematic Reviews and Meta-Analyses (PRISMA) guidelines (15). The protocol was prospectively registered in the PROSPERO database (registration number: CRD420251208049). Study eligibility was determined based on the PICOS criteria, specifying: participants (adults aged 18 years or older), intervention (curcumin supplementation), comparison (placebo group), outcomes (stress, anxiety, and depression), and study design (RCTs).

### Search strategy

We systematically searched PubMed, Scopus, Web of Science Core Collection, and Google Scholar databases from inception to December 2025, without restrictions on language or publication year. The search strategy was developed based on the PICOS framework, including adults aged 18 years or older (population), curcumin supplementation (intervention), placebo (comparison), stress, anxiety, and depression (outcomes), and RCTs (study design). Medical Subject Heading (MeSH) terms and relevant free-text keywords were used and combined with the terms as follows: (“curcumin” OR “curcuminoid” OR “Curcuma” OR “Turmeric” OR “Tumeric” OR “Curcuma longa” OR “C. longa”) AND (“stress” OR “psychological stress” OR “mental health” OR “anxiety” OR “generalized anxiety disorder” OR “depression” OR “depressive symptoms” OR “mood disorder”) AND (“randomized controlled trial” OR “RCT” OR “clinical trial”). To ensure completeness, the reference lists of all eligible articles and related systematic reviews were also manually screened to identify any additional relevant studies.

### Study selection and eligibility criteria

Studies were considered eligible for inclusion in the main analysis if they met all of the following conditions: 1) employed a RCT design; 2) involved adult participants aged over 18 years; 3) reported mental health outcomes, including anxiety, stress, or depression, at both baseline and post-intervention for both the intervention and placebo groups; and 4) evaluated curcumin interventions with a duration of more than 2 weeks.

Studies were excluded if they met any of the following criteria: 1) duplicate publications; 2) lack of a placebo control group; 3) conducted in animals, children, or pregnant or lactating women; 4) non-randomized study designs; or 5) insufficient data on the outcomes of interest.

### Data extraction

Data extraction and study selection were independently performed by two reviewers to ensure accuracy and minimize bias. For each eligible trial, the following information was systematically collected: the surname of the first author, year of publication, participants’ demographic characteristics including age and sex, study setting and duration, trial design, administered curcumin formulation and dosage, sample size per study arm, as well as reported outcome measures with corresponding means and standard deviations (SDs) for anxiety, stress, and depression at both baseline and post-intervention assessments.

### Risk of bias assessment

The methodological quality and potential for bias in the included studies were evaluated using the Cochrane Risk of Bias Tool (16). This instrument examines several domains of bias, including the generation of the random sequence, allocation concealment, blinding of participants and study personnel, blinding of outcome assessment, completeness of outcome data, selective reporting, and other sources of potential bias. Each domain was classified as low risk, some concerns, or high risk of bias. The overall quality of the included studies was ranked as follows: low (if all domains indicated ‘low risk’), moderate (if 1 or more domains indicated ‘some concerns’), and high (if 1 or more domains indicated ‘high risk’). Assessments were conducted independently by two reviewers, with any disagreements resolved through discussion and consensus.

### Certainty of evidence

The quality and confidence in the evidence for each outcome were appraised using the Grading of Recommendations Assessment, Development, and Evaluation (GRADE) framework (17). This approach evaluates the robustness of the evidence across five domains: risk of bias, inconsistency, indirectness, imprecision, and potential for publication bias. Based on these criteria, each outcome was assigned a rating of high, moderate, low, or very low certainty.

### Statistical analysis

Effect sizes were calculated using the mean change and standard deviation (SD) for mental health outcomes such as anxiety, stress, and depression in both the curcumin and placebo groups. When mean change values were not reported, they were estimated from pre- and post-intervention data. Standard errors (SEs), 95% confidence intervals (CIs), or interquartile ranges (IQRs) were converted to SDs when necessary using established statistical methods. (18). When SDs for change scores were not provided, they were estimated using the formula: SD_change = √[(SD_pre^2^ + SD_post^2^) – (2 × r × SD_pre × SD_post)] (19), where a correlation coefficient (*r* = 0.9) was assumed based on previous meta-analyses in similar populations and outcomes, in line with Cochrane Handbook guidance (20, 21).

Pooled effect sizes were reported as standardized mean differences (SMDs) with 95% CIs and were estimated using a random-effects model based on the DerSimonian and Laird method to account for between-study variability. Heterogeneity was evaluated using the I^2^ statistic and Cochrane’s Q test, with significant heterogeneity defined as an I^2^ value greater than 50% or a *P*-value below 0.05 (22–24). Subgroup analyses were conducted to identify potential sources of heterogeneity based on gender (male, female, both), curcumin dosage (< 1 or ≥ 1 g/day), intervention duration (≤ 8 or > 8 weeks), baseline mental health status (healthy, unhealthy), type of intervention (turmeric, unformulated, high-absorption, or nano-curcumin), study quality (low, moderate, high), and study location (Asia, non-Asia).

Sensitivity analyses were carried out using a leave-one-out approach to evaluate how each study affected the overall pooled outcome (25). For crossover trials, appropriate adjustments were applied according to methods recommended by Elbourne et al. (26).

Publication bias was evaluated using funnel plot asymmetry and Begg’s rank correlation test. Additionally, meta-regression and dose–response analyses based on fractional polynomial models were conducted to assess the relationship between curcumin dosage and intervention duration with mental health outcomes (27). All statistical analyses were performed using Stata version 14 (StataCorp, College Station, TX, USA), with *P*-values below 0.05 considered statistically significant.

## Results

### Study selection

A comprehensive database search initially identified 1,413 records. Following the removal of 1,289 duplicate or nonrelevant items, 124 publications proceeded to the title and abstract screening stage. At this phase, 96 articles were excluded for not meeting the inclusion criteria, primarily due to being animal-based investigations, unrelated topics, secondary analyses, or short communications.

Thereafter, 28 full-text manuscripts underwent a detailed eligibility evaluation. Among these, two were excluded as they failed to report the prespecified outcome measures. Ultimately, 26 studies met all eligibility requirements and were included in the quantitative synthesis (28–53), comprising studies focused on depression (25 studies [28–45, 47–53], anxiety (15 studies [28, 30–32, 35, 37–41, 43, 45–47, 50]), and stress (four studies [28, 39, 43, 46]). Figure 1 depicts the PRISMA flow diagram, which systematically summarizes the sequential phases of identification, screening, eligibility appraisal, and final inclusion of studies in the present review.

**Fig. 1:**
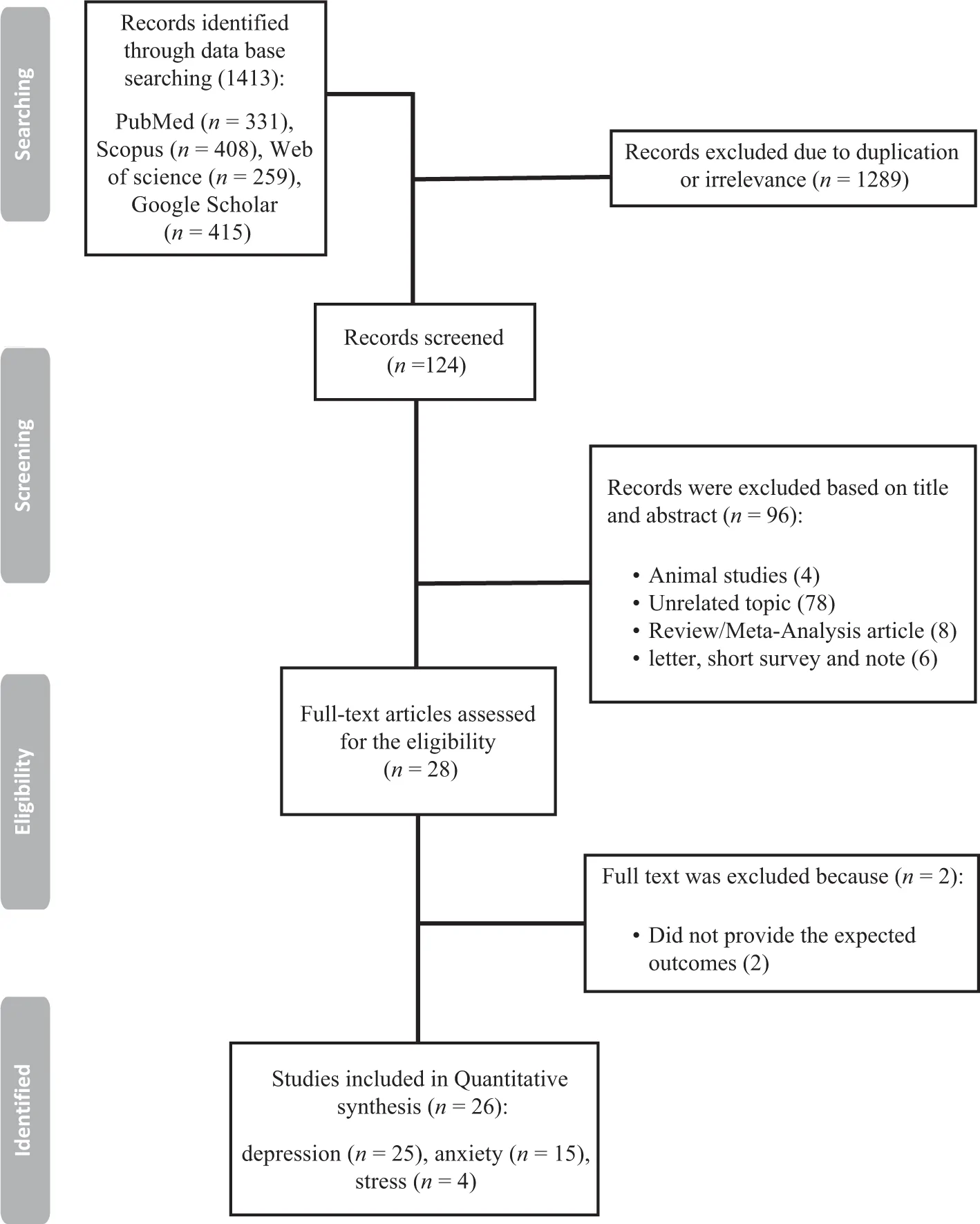
Flow diagram of study selection.

### Characteristics of included studies

Table 1 summarizes the characteristics of the 26 RCTs included in the current systematic review and meta-analysis. The included trials were conducted across several countries, mainly in Iran, followed by Australia, China, Israel, India, the United States, Brazil, Japan, Thailand, and Indonesia, and were published between 2013 and 2025.

**Table 1:** Characteristics of included studies

Author, Year (location)	Study design	Population	Gender	Number (intervention/ control)	Mean age (range)	Duration (weeks)	Intervention group/ control group	Formulation and co-supplementation	Curcumin dosage	Outcome (measures)
Asadi et al., 2019 (Iran)	RCT, DB, parallel	T2DM with diabetic peripheral neuropathy	M/F	40/40	30–60	8	Nano-curcumin/placebo (polysorbate)	Nano-curcumin (Exir Nano Sina Co.); nanoscale formulation containing curcuminoids (72% curcumin, 25% desmethoxycurcumin, and 3% bisdemethoxycurcumin); no additional bioavailability enhancer reported.	80	depression, anxiety, stress (DASS-21)
Bergman et al., 2013 (Israel)	RCT, DB, parallel	Major depressive disorder	M/F	20/20	63.6	5	Curcumin forte + antidepressant medications/placebo + antidepressant medications	Curcumin forte balance (extracts H. Plant, Ra’anana, Israel); containing 330 mg curcumin (97% concentrate), 120 mg ellagic acid (70% pomegranate peel extract), and 50 mg piperine per capsule; co-administered with antidepressant medication.	500	depression, (HAM-D17)
Esmaily et al., 2015 (Iran)	RCT, DB, cross-over	Individuals with obesity	M/F	15/15	38.3	10	Curcumin (C3 complex^®^) + 5 mg bioperine^®^/placebo (5 mg bioperine^®^)	C3 Complex^®^ (Sami Labs Ltd.); curcuminoid mixture (curcumin, demethoxycurcumin, and bisdemethoxycurcumin) co-administered with 5 mg BioPerine^®^ (piperine) to enhance bioavailability; 500 mg C3 Complex^®^ per capsule.	1,000	depression, anxiety (BDI, BAI)
Farshbaf-Khalili et al., 2022 (Iran)	RCT, TPB, parallel	Healthy postmenopausal women	F	28/28	52	8	Curcumin/placebo (microcrystalline cellulose)	Curcumin (Barij Essence Pharmaceutical Co.); standardized turmeric root extract (95%) providing 475 mg curcuminoids.	1,000	depression, anxiety (GCS)
Hosseininasab et al., 2021 (Iran)	RCT, DB, parallel	Chronic schizophrenia	M	29/29	18–60	16	Nanocurcumin + antipsychotic regimen/placebo + antipsychotic regimen	Nanocurcumin (SinaCurcumin; Exir Nano Sina Co.); nanoscale curcumin formulation designed to enhance solubility, dispersion, and oral bioavailability.	160	depression (CDSS)
Kanchanatawan et al., 2018 (Brazil)	RCT, DB, parallel	Patients with major depression	M/F	25/31	18–63	16	Curcumin + antidepressant medications/placebo + antidepressant medications	Curcumin (Government Pharmaceutical Organization [GPO], Bangkok, Thailand); standardized curcuminoids (100%) comprising 77% curcumin, 17% demethoxycurcumin, and 6% bisdemethoxycurcum, co-administered with antidepressant medication.	500–1,500	depression (MADRS)
Kawasaki et al., 2018 (Japan)	RCT, DB, parallel	Healthy participants who regularly drank alcohol	M/F	16/16	20–64	8	Water extract of C. longa/placebo (dextrin)	Water extract of C. longa; hot-water extract standardized to 667 g/kg water extract of C. longa (WEC) and 1.78 g/kg bisacurone; spray-dried formulation with dextrin.	900	depression, anxiety (J-POMS-B)
Kuszewski et al., 2020 (Australia)	RCT, DB, parallel	Older adults with overweight/obesity	M/F	38/36	50–80	16	Curcumin (Longvida^®^)/placebo (maltodextrin)	Longvida^®^ (Blackmores Institute, Sydney, Australia); lipid/solid-lipid-based enhanced-bioavailability formulation, with 400 mg Longvida^®^ providing 80 mg curcumin per capsule.	800	depression (CES-D)
Lopresti et al., 2014 (Australia)	RCT, DB, parallel	Major depressive disorder	M/F	28/28	18–65	8	Curcumin/placebo (cellulose)	BCM-95^®^ (Arjuna Natural Extracts Ltd., Kochi, India); standardized curcuminoid formulation containing 88% total curcuminoids (curcumin, demethoxycurcumin, and bisdemethoxycurcumin) and 7% volatile oils from Curcuma longa rhizomes.	1,000	depression, anxiety (IDS-SR30)
Lopresti et al., 2017 (Australia)	RCT, DB, parallel	Major depressive disorder	M/F	30/31	18–65	12	Curcumin (BCM-95^®^)/placebo (cellulose)	BCM-95^®^ (Dolcas-Biotech LLC, New Jersey, USA); standardized curcuminoid formulation containing 88% total curcuminoids (curcumin, demethoxycurcumin, and bisdemethoxycurcumin) and 7% volatile oils from Curcuma longa rhizomes.	1,000	depression, anxiety (IDS-SR30)
Lopresti et al., 2021 (Australia)	RCT, DB, parallel	Adults with self-reported digestive complaints	M/F	38/39	18–65	8	Curcumin (Curcugen™)/placebo (cellulose)	Curcugen™ (DolCas Biotech LLC); dispersible turmeric-based formulation containing 50% curcuminoids, 1.5% essential oils, and other native turmeric molecules; polar resins enhance curcuminoid self-dispersion and absorption; 500 mg per capsule.	500	depression, anxiety, stress (DASS-21)
Ma et al., 2021 (China)	Open-label controlled trial, parallel	Pulmonary hypertension	M/F	15/15	38.6	12	Curcumin + antidepressant medications/placebo + antidepressant medications	Curcumin, formulation not further specified; co-administered with antidepressant medication; specific physicochemical characteristics not reported.	60–120	depression, anxiety (SDS, SAS)
Mamsharifi et al., 2023 (Iran)	RCT, DB, parallel	Smokers	M/F	35/35	18–50	12	Nano-curcumin/placebo (starch)	Nano-curcumin (Exir Nano Sina Co., Tehran, Iran); nanoscale formulation designed to enhance curcumin solubility, dispersion, and oral bioavailability.	80	depression, anxiety (BDI, BAI)
Miodownik et al., 2019 (Israel)	RCT, DB, parallel	Chronic schizophrenia	M/F	17/16	53.7	24	Curcumin forte + antidepressant medications/placebo + antidepressant medications	Curcumin Forte^®^ (Solgar); containing 95% curcumin and 5% piperine; enhanced-bioavailability formulation, co-administered with antidepressant medication.	3,000	depression (CDSS)
Mojtahedi et al., 2025 (Iran)	RCT, DB, parallel	Migraine patients	M/F	22/22	20–50	8	Nano-curcumin/placebo (paraffin oil)	Nano-curcumin; approximately 100% curcumin with negligible amounts of polysorbate, gelatin, and glycerin; 40 mg nano-curcumin equivalent to approximately 1 g conventional curcumin.	80	depression, anxiety, stress (DASS-21)
Osali et al., 2023 (Iran)	Open-label controlled trial, parallel	Women with metabolic syndrome	F	11/11	50–65	6	Nano-curcumin/placebo	Nano-curcumin; specific physicochemical characteristics not reported.	80	depression (BDI)
Panahi et al., 2014 (Iran)	Open-label controlled trial, parallel	Major depressive disorder	M/F	61/50	40	6	Curcumin (C3 complex^®^) + standard antidepressive therapy/placebo + standard antidepressive therapy	C3 Complex^®^ (Sami Labs Ltd., Bangalore, India); standardized curcuminoid formulation co-administered with 10 mg BioPerine^®^ (piperine) as a bioavailability enhancer, co-administered with antidepressant medication.	1,000	depression, anxiety (HADS)
Pishevar et al., 2025 (Iran)	RCT, DB, parallel	Primiparous women	F	42/43	28	8	Curcumin (curcuma longa extract)/ placebo	Curcumin (Curcuma longa extract; Adineh Co.); 500 mg extract per capsule with unspecified excipients.	500	depression, anxiety (EPDS, PSASRSF)
Saberi-Karimian et al., 2018 (Iran)	RCT, DB, parallel	Metabolic syndrome	M/F	37/36	18–65	6	Phospholipidated curcumin/placebo (lactose and starch)	Absorption-enhanced curcumin; enhanced-bioavailability formulation providing 200 mg pure curcumin/day from 1 g/day curcumin preparation; compared with unformulated curcumin	1,000	depression, anxiety (BDI, BAI)
Sanmukhani et al., 2013 (USA)	Open-label controlled trial, parallel	Major depressive disorder	M/F	18/17	40	6	Curcumin (BCM-95^®^) + 20 mg/day fluoxetine/ placebo + 20 mg/day fluoxetine	BCM-95^®^ (Arjuna Natural Extracts, Kochi, India); standardized curcuminoid formulation containing 88% total curcuminoids (curcumin, demethoxycurcumin, and bisdemethoxycurcumin) and 7% volatile oils from Curcuma longa rhizomes; co-administered with fluoxetine (20 mg/day).	1,000	depression, (HAM-D17)
Setiawati et al., 2017 (Indonesia)	RCT, DB, parallel	Systemic lupus erythematosus	F	10/4	20–59	4	Curcumin (Curcuma xanthorrhiza roxb. extract)/ placebo	Curcuma xanthorrhiza Roxb. extract; botanical curcuminoid-containing powder extract providing 50 mg curcuminoids per capsule.	1,500	depression (BDI)
Shafabakhsh et al., 2020 (Iran)	RCT, DB, parallel	T2DM and coronary heart disease	M/F	25/24	45–85	12	Curcumin/placebo (starch)	Curcumin (Dineh Pharmaceutical Co., Tehran, Iran); formulation not further specified.	1,000	depression, anxiety (BDI, BAI)
Soltani et al., 2024 (Iran)	RCT, DB, parallel	Patients with coronary slow flow phenomenon	M/F	21/21	35–70	12	Nano-curcumin/placebo	Nano-curcumin (Exir Nano Sina Co., Iran); containing a curcuminoid mixture of 79.4% curcumin, 17.6% demethoxycurcumin, and 3% bisdemethoxycurcumin, with polysorbate.	80	depression (BDI)
Sudheeran et al., 2016 (India)	RCT, DB, parallel	Healthy adult	M/F	20/20	33	4	Curcumin/placebo	Curcuma galactomannosides (CGM; Akay Flavours and Aromatics Pvt. Ltd., Kerala, India); enhanced-bioavailability curcumin formulation containing 391 mg total curcuminoids per 1,000 mg CGM (307 mg curcumin, 52.7 mg DMC, 11.3 mg BDMC).	1,000	anxiety, stress (BAI, PSS-14)
Yaikwawong et al., 2024 (Thailand)	RCT, DB, parallel	T2DM	M/F	113/114	≥35	52	Turmeric (Curcuma longa Linn.) rhizomes/ placebo	Turmeric (Curcuma longa Linn.) curcuminoid extract; ethanol-extracted and standardized to 75–85% total curcuminoids; standardized capsules containing 250 mg curcuminoids.	750	depression (PHQ-9)
Yu et al., 2015 (China)	RCT, DB, parallel	Major depressive disorder	M	50/50	31–59	6	Curcumin/placebo (soybean powder)	Curcumin (Shijiazhuang Lvchuan Biological Technology Co., Ltd., China); purity >95%, containing 70% curcumin, 20% demethoxycurcumin, and 10% bisdemethoxycurcumin.	1,000	depression, (HAM-D17)

The sample sizes of the included RCTs ranged from 22 to 227 participants, resulting in a total pooled sample of approximately 1,491 individuals. Participants’ ages ranged from 18 to 85 years, and the duration of interventions varied between 4 and 52 weeks. The majority of the trials adopted a double-blind, parallel-group design.

Regarding the type of intervention, three studies used turmeric (35, 49, 52), seven studies administered unformulated curcumin (31, 34, 38, 40, 46, 50, 53), nine studies used high-absorption curcumin formulations (e.g. BCM-95®, C3 complex®, Curcugen™, or phospholipidated curcumin) (29, 30, 36, 37, 39, 42, 45, 47, 48), and six studies applied nano-curcumin (28, 33, 41, 43, 44, 51). The daily curcumin dose across trials ranged from 60 mg to 3,000 mg.

In terms of mental health status, 17 studies were conducted among psychologically healthy individuals (28, 30–32, 35, 36, 39–41, 43, 44, 46, 47, 49–52), whereas nine studies (29, 33, 34, 37, 38, 42, 45, 48, 53) involved participants with impaired mental health, including those diagnosed with major depressive disorder and schizophrenia. Some of these participants also had comorbid physical illnesses, such as metabolic syndrome (44, 47), migraine (43), systemic lupus erythematosus (49), or type 2 diabetes mellitus (28, 50, 52).

Across the included trials, mental health outcomes were evaluated using a range of standardized and validated psychometric instruments, most frequently the Beck Depression Inventory (BDI), Hamilton Depression Rating Scale (HAM-D17), Montgomery–Åsberg Depression Rating Scale (MADRS), Hospital Anxiety and Depression Scale (HADS), and Beck Anxiety Inventory (BAI), among other established assessment tools.

### Results from quality assessment

The methodological quality of the included RCTs is presented in Table 2. Overall, most studies showed a low risk of bias across the evaluated domains. Random sequence generation and blinding of participants and personnel were adequately reported in almost all trials. Five RCTs (30, 40, 44, 45, 48) were judged to have a high risk of bias in allocation concealment. For blinding of outcome assessment, most studies were rated as low risk, except for a few studies (40, 44, 45, 48), which were assessed as high risk. In terms of incomplete outcome data, nearly all RCTs showed low risk, with some studies (40, 44, 45) presenting some concerns. Selective outcome reporting was generally well controlled, with most studies rated as low risk and only a few RCTs showing some concerns (29, 30, 53). Finally, regarding other potential threats to validity, most RCTs were considered low risk except for a few RCTs (30, 40, 44, 45, 48), which were judged to have a high risk of bias.

**Table 2:** Results of risk of bias assessment for randomized clinical trials included in the current meta-analysis

Author, Year	Sequence generation	Allocation concealment	Blinding of participants and personnel	Blinding of outcome assessment	Incomplete outcome data	Selective outcome reporting	Other potential threats to validity	Overall risk of bias
Asadi et al., 2019	L	S	L	L	L	L	S	M
Bergman et al., 2013	L	L	L	L	L	S	S	M
Esmaily et al., 2015	H	S	L	L	L	S	H	H
Farshbaf-Khalili et al., 2022	L	L	L	L	L	L	L	L
Hosseininasab et al., 2021	S	L	L	L	L	L	S	M
Kanchanatawan et al., 2018	L	L	L	L	L	L	L	L
Kawasaki et al., 2018	L	L	L	L	L	L	L	L
Kuszewski et al., 2020	L	L	L	L	L	L	L	L
Lopresti et al., 2014	L	L	L	L	L	L	L	L
Lopresti et al., 2017	L	L	L	L	L	L	L	L
Lopresti et al., 2021	L	L	L	L	L	L	L	L
Ma et al., 2021	H	H	H	H	S	L	H	H
Mamsharifi et al., 2023	L	L	L	L	L	L	L	L
Miodownik et al., 2019	L	L	L	L	L	L	L	L
Mojtahedi et al., 2025	L	L	L	L	L	L	L	L
Osali et al., 2023	H	H	H	H	S	L	H	H
Panahi et al., 2014	H	H	H	H	S	L	H	H
Pishevar et al., 2025	L	L	L	L	L	L	L	L
Saberi-Karimian et al., 2018	L	L	L	L	L	L	L	L
Sanmukhani et al., 2013	H	H	H	H	L	L	H	H
Setiawati et al., 2017	S	S	L	L	L	L	S	M
Shafabakhsh et al., 2020	L	L	L	L	L	L	L	L
Soltani et al., 2024	L	L	L	L	L	L	L	L
Sudheeran et al., 2016	L	L	L	L	L	L	L	L
Yaikwawong et al., 2024	L	L	L	L	L	L	L	L
Yu et al., 2015	L	S	L	S	L	S	S	M

The overall quality of the included studies was as follows: 16 RCTs were considered high quality (31, 32, 34–39, 41–43, 46, 47, 50–52), five RCTs were of moderate quality (28, 29, 33, 49, 53), and five RCTs were of low quality (30, 40, 44, 45, 48).

### Meta-analysis and subgroup analysis

The pooled meta-analysis demonstrated that curcumin supplementation exerted a significant beneficial effect on mental health outcomes, leading to reductions in depression (SMD = –1.53; 95% CI: –2.05 to –1.02; *I^2^* = 94.8%; Fig. 2), anxiety (SMD = –1.67; 95% CI: –2.42 to –0.93; *I^2^* = 95.7%; Fig. 3), and stress (SMD = –1.71; 95% CI: –3.37 to –0.06; *P* = 0.042; *I^2^* = 96.3%; Fig. 4) across the included RCTs.

**Fig. 2:**
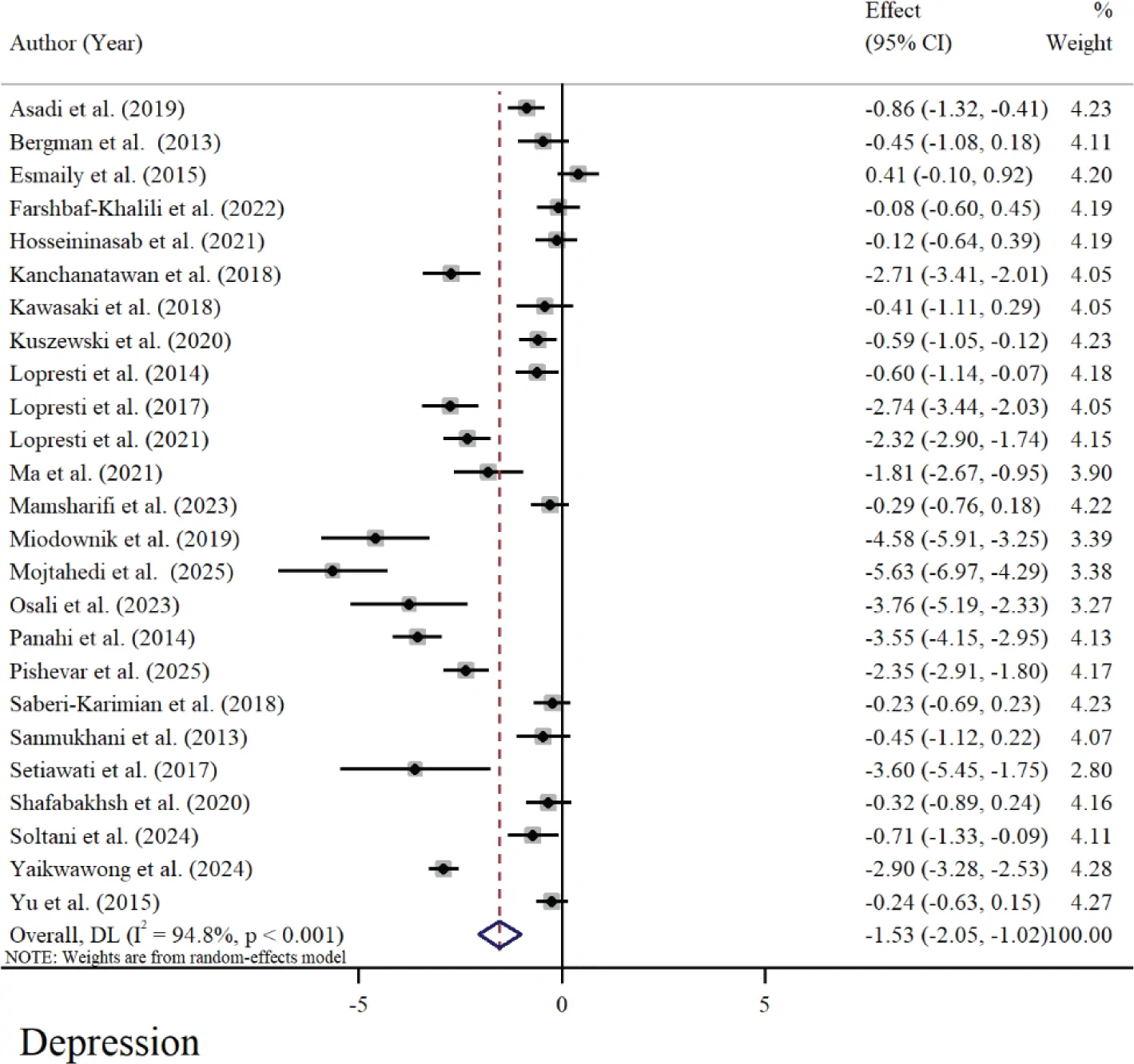
Forest plots for the effect of curcumin supplementation on depression. Horizontal lines represent 95% CIs. Diamonds represent pooled estimates from random-effects analysis. SMD, standardized mean difference; CI, confidence interval.

**Fig. 3:**
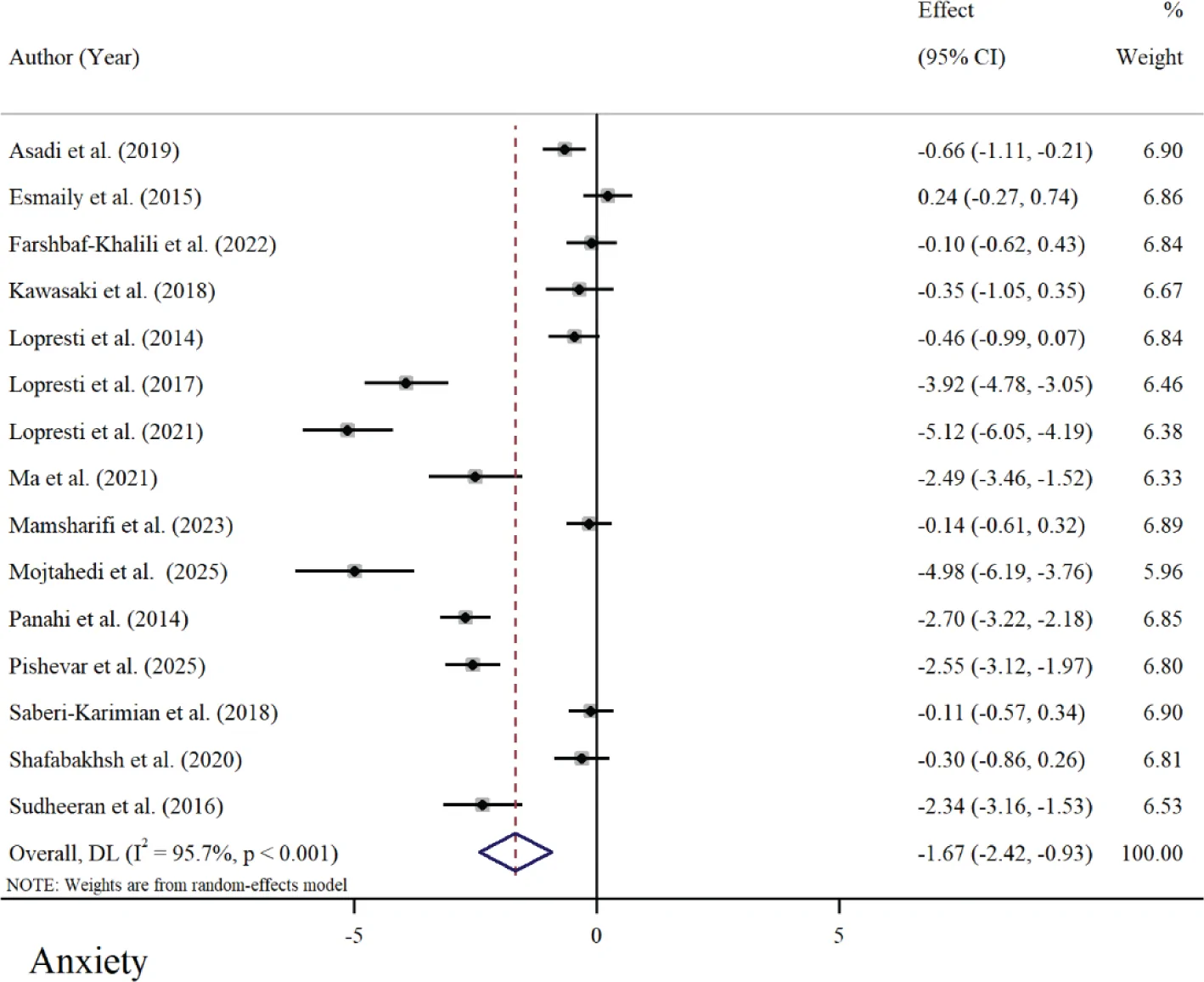
Forest plots for the effect of curcumin supplementation on anxiety. Horizontal lines represent 95% CIs. Diamonds represent pooled estimates from random-effects analysis. SMD, standardized mean difference; CI, confidence interval.

**Fig. 4:**
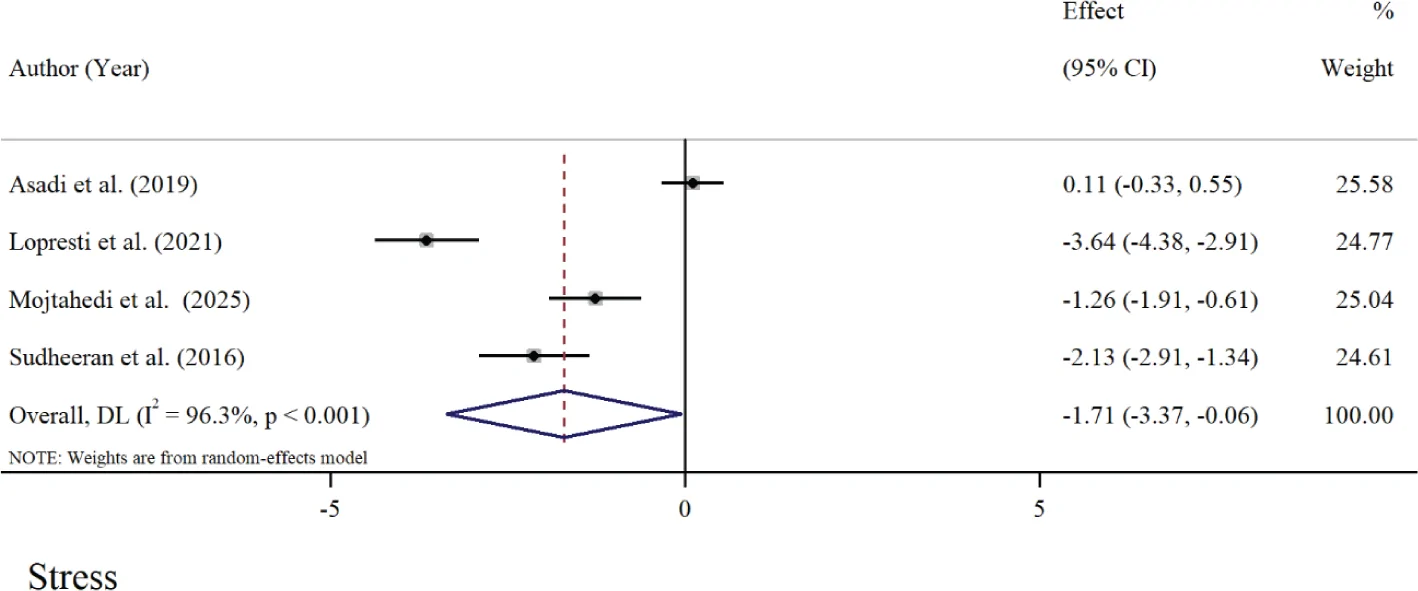
Forest plots for the effect of curcumin supplementation on stress. Horizontal lines represent 95% CIs. Diamonds represent pooled estimates from random-effects analysis. SMD, standardized mean difference; CI, confidence interval.

Table 3 presents the subgroup analyses evaluating the effects of curcumin supplementation on mental health outcomes. For depression, a significant difference between subgroups was observed only for gender, with larger effect sizes in female participants and no significant effect in males. Differences in magnitude were also evident across intervention types, with turmeric showing the largest effect, followed by high-absorption curcumin, while unformulated and nano-curcumin had smaller effects. Although effect sizes varied across study quality categories, significant effects were observed in all (low, moderate, and high risk of bias), and the between-subgroup difference was not statistically significant. No meaningful differences in effect size were observed across other subgroups.

**Table 3:** Subgroup analysis to assess the effect of curcumin on mental health

Variable	Number of effect sizes	SMD (95% CI)	*P* ^1^	*I*2 (%)^2^	*P*-heterogeneity^3^	*P*-between subgroup heterogeneity^4^
**Depression**						
**Overall**	25	−1.53 (−2.05, −1.02)	< 0.001	94.8	< 0.001	–
***Gender***						< 0.001
Male	2	−0.19 (−0.50, 0.11)	0.218	0.0	0.726	
Female	4	−1.40 (−1.76, −1.04)	< 0.001	94.2	< 0.001	
Both	19	−1.25 (−1.38, −1.11)	< 0.001	95.1	< 0.001	
***Dosage (g/day)***						0.335
< 1	13	−1.36 (−1.52, −1.20)	< 0.001	94.4	< 0.001	
≥ 1	12	−0.85 (−1.02, −0.68)	< 0.001	95.1	< 0.001	
***Duration (weeks)***						0.778
≤ 8	14	−1.05 (−1.21, −0.89)	< 0.001	94.3	< 0.001	
> 8	11	−1.19 (−1.36, −1.03)	< 0.001	95.7	< 0.001	
***Mental health status***						0.945
Healthy	16	−1.11 (−1.25, −0.97)	< 0.001	94.7	< 0.001	
Unhealthy	9	−1.15 (−1.35, −0.95)	< 0.001	95.4	< 0.001	
***Type of intervention***						0.138
Turmeric	3	−2.38 (−2.66, −2.10)	< 0.001	92.4	< 0.001	
Unformulated curcumin	7	−0.64 (−0.87, −0.42)	< 0.001	90.1	< 0.001	
High absorption curcumin	9	−1.11 (−1.31, −0.92)	< 0.001	95.7	< 0.001	
Nano-curcumin	6	−0.76 (−1.01, −0.52)	< 0.001	93.5	< 0.001	
***Location***						0.610
Asia	19	−1.05 (−1.18, −0.92)	< 0.001	95.4	< 0.001	
Non-Asia	6	−1.37 (−1.61, −1.13)	< 0.001	92.3	< 0.001	
***Study quality (risk of bias)***						0.124
Low risk	15	−3.13 (−4.21, −2.04)	< 0.001	97.1	< 0.001	
Moderate risk	5	−2.40 (−4.17, −0.66)	0.007	92.7	< 0.001	
High risk	5	−3.99 (−7.19, −0.78)	0.015	96.8	< 0.001	
**Anxiety**						
**Overall**	15	−1.67 (−2.42, −0.93)	< 0.001	95.7	< 0.001	–
***Gender***						0.872
Male	–	–	–	–	–	
Female	2	−1.21 (−1.60, −0.82)	< 0.001	97.4	< 0.001	
Both	13	−1.03 (−1.20, −0.87)	< 0.001	95.8	< 0.001	
***Dosage (g/day)***						0.490
> 1	7	−1.38 (−1.62, −1.14)	< 0.001	96.3	< 0.001	
≥ 1	8	−0.87 (−1.03, −0.63)	< 0.001	95.4	< 0.001	
***Duration (weeks)***						0.426
≤ 8	10	−1.27 (−1.46, −1.09)	< 0.001	95.9	< 0.001	
> 8	5	−0.61 (−0.88, −0.34)	< 0.001	95.3	< 0.001	
***Mental health status***						0.242
Healthy	12	−0.83 (−1.00, −0.66)	< 0.001	95.3	< 0.001	
Unhealthy	3	−1.96 (−2.30, −1.62)	< 0.001	96.6	< 0.001	
**Type of intervention**						0.825
Turmeric	2	−1.65 (−2.10, −1.21)	< 0.001	95.6	< 0.001	
Unformulated curcumin	5	−0.70 (−0.98, −0.42)	< 0.001	89.1	< 0.001	
High absorption curcumin	5	−1.40 (−1.66, −1.14)	< 0.001	97.9	< 0.001	
Nano-curcumin	3	−0.71 (−1.02, −0.90)	< 0.001	96.2	< 0.001	
***Location***						0.274
Asia	12	−0.89 (−1.05, −0.72)	< 0.001	94.5	< 0.001	
Non- Asia	3	−2.10 (−2.51, −1.70)	< 0.001	97.5	< 0.001	
***Study quality (risk of bias)***						0.112
Low risk	11	−2.47 (−3.39, −1.55)	< 0.001	95.7	< 0.001	
Moderate risk	1	−1.10 (−1.83, −0.36)	0.003	–	–	
High risk	3	−3.28 (−6.69, 0.13)	0.060	96.1	< 0.001	
Stress*						
**Overall**	4	−1.71 (−3.37, −0.06)	0.042	96.3	< 0.001	–

1 Refers to the SMD (95% CI).

2 Indicates between-study heterogeneity (as percentage).

3 Obtained from the Q-test.

4 Obtained from the fixed-effect model.

* Subgroup analysis was not performed due to the limited number of included studies.

For anxiety, larger effect sizes were observed in studies using lower dosages (< 1 g/day) and shorter intervention durations (≤ 8 weeks), as well as in participants with psychological disorders compared to healthy individuals. Variation was also observed across intervention types, with turmeric and high-absorption formulations showing greater effects. In subgroup analysis based on study quality, significant effects were observed in low- and moderate-risk studies, whereas no statistically significant effect was found in high-risk studies; however, the between-subgroup difference was not statistically significant. Other subgroup categories showed no significant differences.

For stress, only the overall analysis showed a significant reduction, and no subgroup analyses were conducted due to the limited number of included studies.

### Sensitivity analysis

Sensitivity analyses were performed by sequentially omitting each study to evaluate the influence of individual trials on the overall effect size. The pooled estimates remained generally stable for depression (95% CI: –2.13 to –0.88) and anxiety (95% CI: –2.59 to –0.73), indicating that no single study unduly influenced the combined results. For stress (95% CI: –4.24 to 0.53), minor fluctuations were observed following the omission of individual studies; however, these changes did not materially affect the overall interpretation, and the pooled effect remained directionally consistent toward stress reduction.

### Publication bias and trim-and-fill analysis

Publication bias was evaluated using Begg’s rank correlation test in conjunction with visual inspection of funnel plots (Supplementary Figure 1). The funnel plots showed a slight asymmetry for the included trials, suggesting the possibility of publication bias for some outcomes.

According to Begg’s test results, evidence of potential publication bias was observed for anxiety (*P* = 0.021), whereas no statistically significant bias was detected for depression (*P* = 0.607) or stress (*P* = 0.157). Given the statistically significant result identified for anxiety, a trim-and-fill analysis was subsequently conducted to further assess the robustness of the findings. This analysis did not identify any missing (imputed) studies, and the pooled effect sizes for all mental health outcomes remained materially unchanged after adjustment. It is important to note that the number of studies included in the analysis of stress was limited, which may have reduced the statistical power to detect potential publication bias for this outcome.

### Meta-regression and nonlinear dose–response and time-response analyses

The meta-regression analyses showed that no significant linear relationships were found between curcumin dosage or intervention duration and changes in depression (β = −0.002, *P* = 0.094; duration: β = −0.061, *P* = 0.450), anxiety (dose: β = 0.003, *P* = 0.386; duration: β = 0.274, *P* = 0.834), or stress (dose: β = −0.143, *P* = 0.214; duration: β = 0.287, *P* = 0.186).

As illustrated in Fig. 5, the nonlinear dose–response analysis revealed that curcumin dosage exhibited a significant association with depression, indicating that higher doses (> 1.5 g/day) tended to be associated with greater improvements in depressive symptoms (*P*-nonlinearity = 0.047). In contrast, no significant nonlinear associations were observed for anxiety (*P*-nonlinearity = 0.176) or stress (*P*-nonlinearity = 0.355). Similarly, intervention duration was not significantly related to changes in depression (*P*-nonlinearity = 0.274), anxiety (*P*-nonlinearity = 0.938), or stress (*P*-nonlinearity = 0.838). Although a nonlinear dose–response curve or linear association was fitted for stress, the results should be interpreted with caution due to the limited number of included studies, which restricts the reliability of the model estimates.

**Fig. 5:**
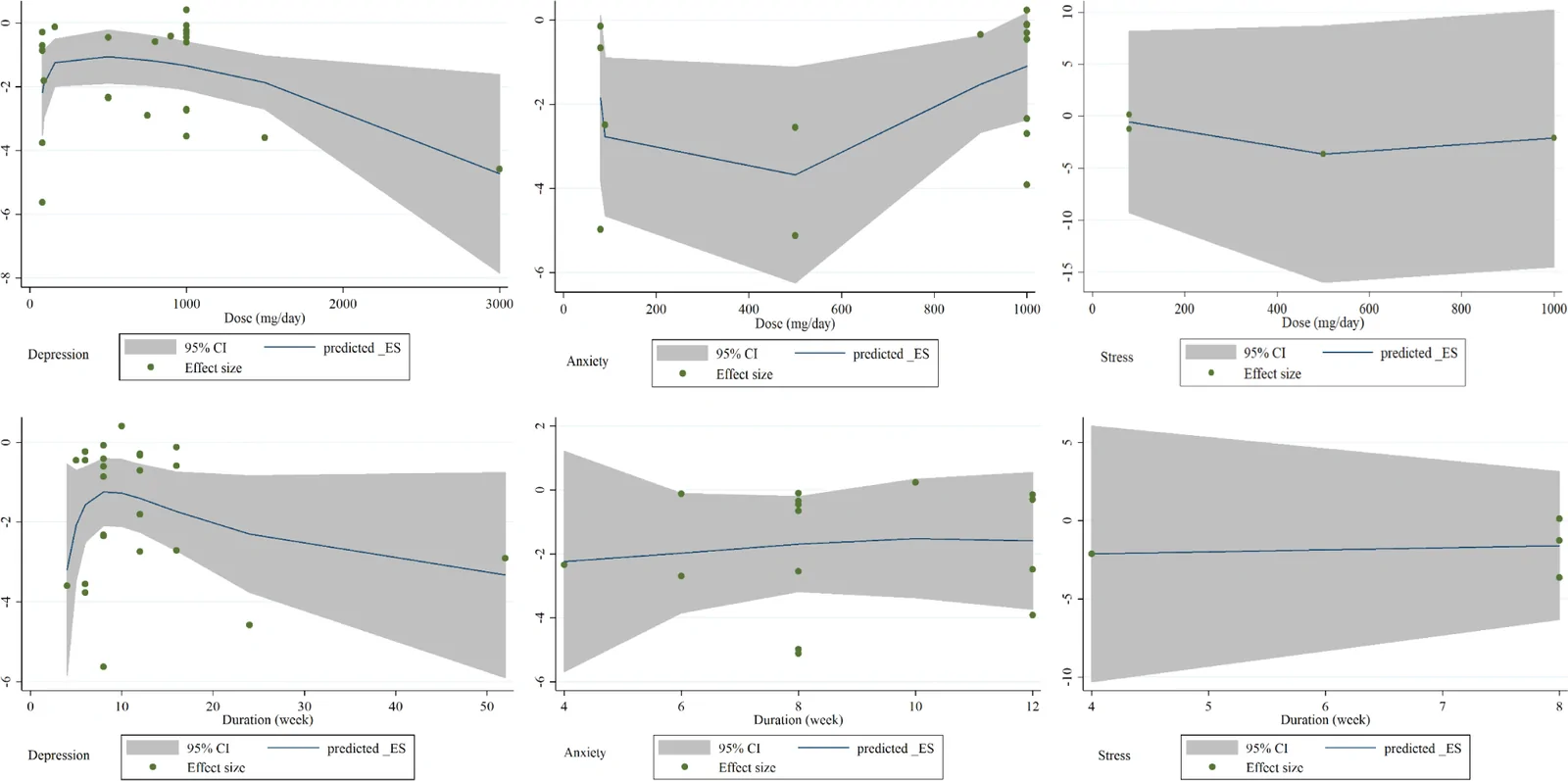
Dose–response relations between curcumin supplementation dosage (mg/day) and duration of intervention (week) with absolute (unstandardized) mean differences of the outcomes in nonlinear fashion.

### Grading of evidence

The quality of evidence for the effects of curcumin supplementation on mental health outcomes was evaluated using the GRADE framework. According to the evidence profiles (Supplementary Table 1), the certainty of evidence was rated as moderate for depression and stress, and low for anxiety. The main reasons for downgrading were between-study inconsistency observed for depression, stress, and anxiety, along with potential publication bias for anxiety.

## Discussion

This meta-analysis comprehensively evaluated the effects of curcumin supplementation on psychological outcomes and demonstrated overall beneficial effects across a broad range of populations and study designs. Curcumin intake was associated with significant reductions in depressive, anxiety, and stress symptoms relative to placebo, supporting its potential as an effective adjunctive or preventive strategy for improving mental well-being. The beneficial effects remained robust in sensitivity analyses, indicating that no single study disproportionately influenced the pooled estimates. Furthermore, meta-regression and dose–response analyses suggested a nonlinear association between curcumin dosage and depressive symptoms, with higher doses generally linked to greater improvements, while intervention duration showed no significant relationship with psychological outcomes. Collectively, these results highlight the psychotropic potential of curcumin and suggest that both dosage and participant characteristics may modulate its therapeutic efficacy.

Importantly, statistical significance should not be interpreted as evidence of clinical significance. The included trials used different validated instruments to assess depression, anxiety, and stress, each with its own scale properties. Because the pooled effects were expressed as SMDs, the observed estimates cannot be directly translated into clinically meaningful changes on individual scales. Therefore, although curcumin supplementation was associated with statistically significant improvements, the extent to which these changes represent clinically meaningful benefits for patients remains uncertain.

Previous meta-analyses investigating the psychological effects of curcumin suggested potential benefits for depression and anxiety (13, 14); however, their conclusions were constrained by methodological and analytical limitations. Most included a small number of trials with short intervention durations, exhibited substantial heterogeneity, and focused narrowly on selected outcomes without examining stress or exploring potential dose–response patterns. The present meta-analysis advances the existing evidence by integrating a larger and more diverse set of RCTs, encompassing all major psychological domains, and by employing rigorous subgroup and dose–response analyses. This comprehensive approach provides a more nuanced and reliable assessment of curcumin’s overall impact on mental health.

Despite the overall positive effects observed in the present meta-analysis, findings across individual RCTs were not entirely consistent. Several studies reported no significant changes in psychological outcomes following curcumin supplementation (30, 31, 47). The heterogeneity in results may be attributed to multiple methodological and biological factors. First, differences in curcumin formulations and bioavailability may partly account for the variability in treatment effects observed across the included studies, as these factors can markedly influence absorption, systemic exposure, and overall therapeutic efficacy. Second, variations in dosage and intervention duration across studies may have influenced the magnitude of effect, with shorter trials and moderate doses often yielding more pronounced benefits. Third, participant characteristics, including baseline psychological status, sex, and presence of comorbidities, may have moderated treatment responses, as greater improvements were observed among women and participants with existing depressive or anxiety disorders. Additionally, differences in study quality, sample size, and assessment tools might explain the observed heterogeneity in effect sizes across trials.

Several neurobiological processes have been proposed to underlie the psychotropic properties of curcumin. Experimental and preclinical evidence indicates that curcumin may modulate monoaminergic neurotransmission by increasing serotonin and dopamine availability, potentially through inhibition of monoamine oxidase (MAO) activity while also enhancing brain-derived neurotrophic factor (BDNF) expression and signaling, thereby promoting neuroplasticity and neuronal resilience (54). In parallel, its potent antioxidant and anti-inflammatory activities may reduce peripheral and central oxidative stress and neuroinflammation, which are increasingly implicated in the pathophysiology of depression, anxiety, and chronic psychological stress. Curcumin may also attenuate hyperactivity of the hypothalamic–pituitary–adrenal (HPA) axis and reduce cortisol secretion, potentially contributing to improved regulation of the stress response (10). Moreover, modulation of the GABAergic and endocannabinoid systems has been proposed as an additional mechanism through which curcumin may exert anxiolytic, antidepressant, and mood-regulating effects (10). Nevertheless, these mechanistic pathways are supported predominantly by experimental and preclinical evidence and should therefore be interpreted as biologically plausible mechanisms rather than definitive causal pathways established in humans.

Several neurobiological processes are proposed to explain the psychotropic properties of curcumin. Experimental and preclinical evidence indicates that curcumin modulates monoaminergic neurotransmission by increasing serotonin and dopamine concentrations through the inhibition of MAO activity and the enhancement of BDNF expression (54). Its potent antioxidant and anti-inflammatory actions are believed to mitigate both peripheral and central inflammation. Furthermore, curcumin may attenuate the hyperactivity of the HPA axis, resulting in reduced cortisol secretion and improved regulation of the stress response. Collectively, these biological effects contribute to enhanced neuronal plasticity and greater resilience to psychological stress. Curcumin may also influence the endocannabinoid and GABAergic systems, which could further support its anxiolytic, antidepressant, and mood-stabilizing effects (10).

Adjunctive use of curcumin alongside standard psychotropic treatment warrants particular consideration. Several included trials evaluated curcumin as an add-on to established psychiatric therapies, including antidepressant or antipsychotic medications. Some included studies (29, 34, 45) administered curcumin in addition to standard antidepressant treatment, while one study (33) evaluated nanocurcumin as an adjunct to an antipsychotic regimen. In these trials, the control groups also received the corresponding standard treatment, allowing the observed between-group differences to be interpreted as the additional effect associated with curcumin supplementation. Collectively, these studies provide supportive evidence that curcumin may offer potential additional benefits when used alongside conventional psychotropic therapy. However, differences in populations, psychiatric diagnoses, background medications, curcumin formulations, and outcome measures, together with the limited number of adjunctive trials, preclude firm conclusions regarding the magnitude of this incremental benefit.

This meta-analysis has several methodological and analytical strengths that enhance the credibility of its findings. It systematically integrates evidence from RCTs on curcumin supplementation and psychological outcomes, incorporating diverse populations across various regions and clinical conditions, thereby improving the generalizability of results. Methodological rigor was ensured through adherence to PRISMA guidelines, PROSPERO registration, standardized data handling, and evaluation of evidence certainty using the GRADE framework. In addition, comprehensive subgroup, sensitivity, and dose–response analyses along with linear meta-regression allowed identification of potential moderators. Careful assessment of publication bias using multiple statistical and graphical methods further strengthened the transparency and reliability of the conclusions.

Nevertheless, several limitations should be considered when interpreting these findings. The presence of substantial heterogeneity suggests considerable methodological and biological variability across studies, including differences in design, dosage, intervention duration, curcumin formulation, and participant characteristics. The overall certainty of evidence was graded as low to very low, primarily due to inconsistency and potential publication bias, particularly in outcomes related to anxiety. Moreover, the predominance of trials conducted in Asian populations may restrict the generalizability of the findings to Western or other populations with different genetic, dietary, and cultural backgrounds. Many included RCTs also featured relatively small sample sizes, short intervention durations, and reliance on self-reported psychological measures, which may introduce subjectivity and measurement bias. Moreover, the absence of objective biological parameters, such as neuroinflammatory biomarkers and neuroimaging measures, limits the ability to corroborate the observed psychological effects and may reduce confidence in their biological interpretation. Differences in curcumin formulations and co-supplementation strategies across trials may also have contributed to between-study heterogeneity although co-supplemented components were generally matched between intervention and control groups when applicable. Finally, incomplete reporting of treatment adherence and adverse events, along with the limited availability of long-term follow-up data, constrains firm conclusions regarding the durability and safety of curcumin’s effects.

## Conclusion

In summary, this updated meta-analysis strengthens and extends previous evidence suggesting that curcumin supplementation may yield meaningful reductions in symptoms of depression, anxiety, and stress. Although the overall certainty of evidence remains limited by heterogeneity and methodological limitations, curcumin appears to be a promising and generally well-tolerated adjunctive approach for improving mental well-being. Nevertheless, future large-scale, high-quality RCTs with standardized formulations, optimized dosages, and longer follow-up durations are warranted to confirm these effects and to better establish the efficacy and safety profile of curcumin in mental health management.

## Supplementary Material



## Data Availability

The datasets analyzed during the current study are available from the corresponding author upon reasonable request.
