# Supplementary material for: Effects of curcumin on depression, anxiety, and stress symptoms in adults: a GRADE-assessed systematic review and dose–response meta-analysis
Source: Food Nutr Res. 2026 Sep 24;70:10.29219/fnr.v70.14735. doi: 10.29219/fnr.v70.14735 (PMC13653422; doi:10.29219/fnr.v70.14735)
Supplement: Supplementary file 1 [file FNR-70-14735-s1.docx]

**Supplementary Figure 1.** Funnel plots for analysis publication bias.

| **Supplementary Table 1.** GRADE profile of the effect of curcumin supplementation and mental health***.** | | | | | | |
| --- | --- | --- | --- | --- | --- | --- |
| **Outcomes** | **Risk of bias** | **Inconsistency** | **Indirectness** | **Imprecision** | **Publication bias** | **Quality of evidence** |
| **Depression** | No serious limitations | Serious limitations | No serious limitations | No serious limitations | No serious limitations | ⊕⊕⊕◯ Moderate |
| **Anxiety** | No serious limitations | Serious limitations | No serious limitations | No serious limitations | Serious  limitations | ⊕⊕◯◯ Low |
| **Stress** | No serious limitations | Serious limitations | No serious limitations | No serious limitations | No serious limitations | ⊕⊕⊕◯ Moderate |
| *Downgrading was applied when concerns were identified, including methodological flaws (risk of bias), substantial heterogeneity across studies (inconsistency), differences in population, intervention, or outcomes (indirectness), wide confidence intervals or small sample sizes (imprecision), and evidence of publication bias or funnel plot asymmetry (publication bias). The overall certainty of evidence was determined based on the total number of downgrades across these domains. | | | | | | |
